# The Fragile X Protein Family in Amyotrophic Lateral Sclerosis

**DOI:** 10.1007/s12035-023-03330-x

**Published:** 2023-03-29

**Authors:** Sarah Mueller, Lorena Decker, Sonja Menge, Albert C. Ludolph, Axel Freischmidt

**Affiliations:** 1 Department of Neurology, Ulm University, Albert-Einstein-Allee 11, 89081 Ulm, Germany; 2 German Center For Neurodegenerative Diseases (DZNE) Ulm, Ulm, Germany

**Keywords:** Neurodegenerative disease, Amyotrophic lateral sclerosis ALS, FMR1 FMRP, FXR1 FXR1P, FXR2 FXR2P, Protein aggregation

## Abstract

The fragile X protein (FXP) family comprises the multifunctional RNA-binding proteins FMR1, FXR1, and FXR2 that play an important role in RNA metabolism and regulation of translation, but also in DNA damage and cellular stress responses, mitochondrial organization, and more. FMR1 is well known for its implication in neurodevelopmental diseases. Recent evidence suggests substantial contribution of this protein family to amyotrophic lateral sclerosis (ALS) pathogenesis. ALS is a highly heterogeneous neurodegenerative disease with multiple genetic and unclear environmental causes and very limited treatment options. The loss of motoneurons in ALS is still poorly understood, especially because pathogenic mechanisms are often restricted to patients with mutations in specific causative genes. Identification of converging disease mechanisms evident in most patients and suitable for therapeutic intervention is therefore of high importance. Recently, deregulation of the FXPs has been linked to pathogenic processes in different types of ALS. Strikingly, in many cases, available data points towards loss of expression and/or function of the FXPs early in the disease, or even at the presymptomatic state. In this review, we briefly introduce the FXPs and summarize available data about these proteins in ALS. This includes their relation to TDP-43, FUS, and ALS-related miRNAs, as well as their possible contribution to pathogenic protein aggregation and defective RNA editing. Furthermore, open questions that need to be addressed before definitively judging suitability of these proteins as novel therapeutic targets are discussed.

## Introduction

The heterogeneous neurodegenerative disease amyotrophic lateral sclerosis (ALS) is characterized by the progressive loss of neurons of the motor system leading to muscular atrophy, weakness, paralysis, and, ultimately, death by respiratory failure. A few percent of the patients can be explained by monogenic inheritance of pathogenic variants in more than two dozen ALS genes (fALS), with *C9orf72*, *SOD1*, *FUS*, and *TARDBP* being the most frequent, at least in populations of European ancestry. However, the vast majority of patients are sporadic (sALS), but, here too, increasing evidence points towards the involvement of genetics and a polygenic architecture of the disease. Most likely, although poorly understood, a complex and heterogeneous interplay of multiple known and unknown genetic and environmental risk factors is responsible for most sALS cases. There is still no cure for ALS, and limited treatment options prolong life expectancy just by a few months. Despite extensive research, molecular mechanisms responsible for the death of motoneurons are not well understood. This is mainly due to the high heterogeneity of cellular defects associated with different ALS genes, and frequent failure to detect pathogenic pathways in patients with another genetic cause and/or in sporadic patients. Nevertheless, some pathogenic processes are shared among most, if not all, ALS patients. Therefore, it is still a matter of debate if different genetic causes should be considered as diverse diseases with a similar outcome, or if different primum movens converge into a single or few pathogenic cascade(s) driving onset and progression of the disease. So far, both hypotheses are supported by solid bodies of evidence, and answering this question is of high importance for better understanding this devastating disease, the development of disease modifying treatments, and the stratification of patients in clinical trials [1, 2].

In this review, we summarize available evidence for an involvement of the fragile X protein (FXP) family, comprising FMR1, FXR1, and FXR2, in ALS pathogenesis. This protein family, and especially FMR1, is famous for their roles in autism spectrum disorders (ASDs) including the fragile X syndrome (FXS), and related diseases reviewed elsewhere. However, recent studies implicate these proteins in certain types of cancer, mental illnesses, and neurodegenerative diseases [3]. Although we are far from understanding their role in ALS, available evidence indicates contribution of this protein family to the disease that may be largely independent of the underlying cause. From a functional point of view, the FXPs have the potential to link key events of ALS pathogenesis, such as hyperexcitability of motoneurons, RNA dysmetabolism, mitochondrial dysfunction, axonal transport and synaptic integrity, and impaired protein homeostasis and protein aggregation. If true, therapeutic approaches aiming at the FXPs may not cure the disease, but may be beneficial for the vast majority, if not all, ALS patients.

## The FXP Family

### Structure

The FXPs constitute a small family of RNA-binding proteins (RBPs) comprising only three members, FMR1 (or FMRP), FXR1 (or FXR1P), and FXR2 (or FXR2P). These proteins are highly homologous to each other, especially in the N-terminal half, while more variation is found in the C-terminal part [3]. Evolutionary, FXPs are highly conserved and most organisms higher than insects encode all three family members in their genomes.

**Fig. 1:**
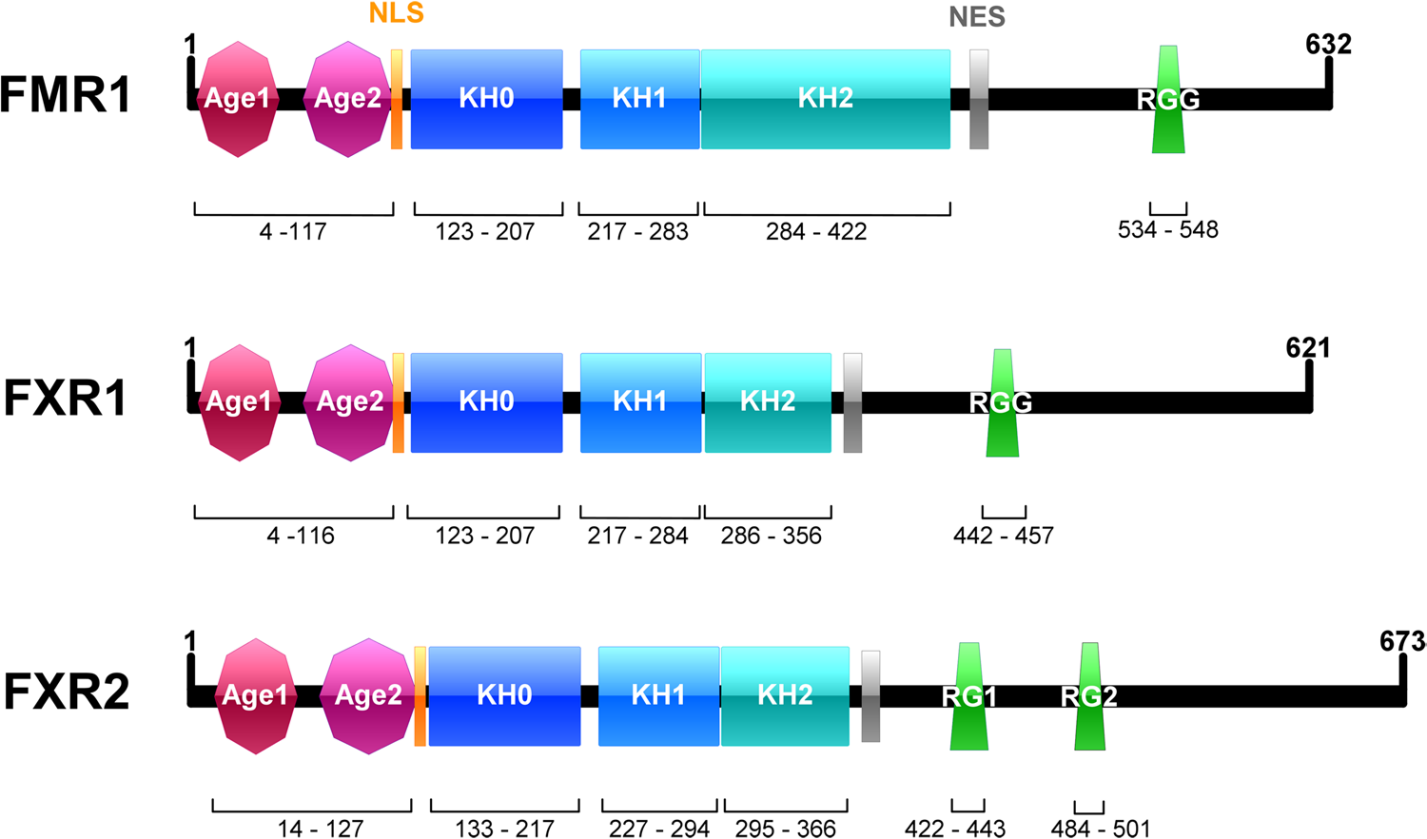
Domains of the fragile X protein family. Each member of the fragile X protein family comprises a tandem agenet-like domain (Age1 and Age2) at the N-terminus, a nuclear localization signal (NLS) as well as a nuclear export signal (NES), three RNA-binding K homology domains (KH0, KH1 and KH2), and a mostly disordered C-terminus that harbours repeats of RNA-binding RGG motifs (in FMR1 and FXR1, or two separate variations of RGG motifs in FXR2, indicated as RG1 and RG2). Canonical isoforms are shown and amino acid positions of the respective domains according to InterPro [10] are indicated. RG1 and RG2 motifs of FXR2 were defined in [6]

The functional domains of the FXP family include a tandem Agenet-like domain (TAD) at the N-terminus followed by a nuclear localization signal (NLS), three RNA-binding K-homology (KH) domains, a nuclear export signal (NES), and a mostly disordered C-terminus comprising RNA-binding RGG motifs, or variations thereof (Fig. 1) [3, 4]. The function of the TAD is unknown, but it is structurally related to TUDOR domains known to recognize and interact with methylated arginines and/or lysines of proteins [5]. Indeed, the TAD of FMR1, but not of FXR1 and FXR2 [6], has been shown to directly interact with the C-terminal region of FUS [7, 8] comprising methylated RGG motifs [9]. The NLS and NES facilitate shuttling between nucleus and cytoplasm, whereby all three FXPs are predominantly found in the cytoplasm. The KH domains as well as the RGG motifs are responsible for the RNA-binding properties of these proteins. However, despite numerous studies mainly focusing on FMR1, sequence and/or structural features of RNAs binding to the FXPs are still poorly understood, and different methodologies and cell types used for target mRNA identification may strongly affect the results. Nevertheless, for FMR1, target mRNAs appear to be mostly related to cellular signaling, development and function of axons, dendrites, and synapses, the microtubule cytoskeleton and RNA transport, and transcriptional and epigenetic regulation of gene expression. While the three KH domains weakly interact with single-stranded RNAs and most likely require specific secondary structures and/or additional RBPs/factors for binding, the RGG domains of the FXPs have been repeatedly, but not consistently, shown to associate with RNA G quadruplexes [3, 4]. Interestingly, a recent study found that the RGG domains of the FXPs directly interact with a specific subset of mature microRNAs (miRNAs) deregulated in ALS, but possible functions have not been elucidated yet [6].

### Function

The FXPs are multifunctional proteins ubiquitously expressed in virtually all tissues and cell types, including the CNS [11]. Most of our knowledge of FXP functions is derived from studying FMR1 in the context of ASDs. As expected from the similar structures and, at least in part, high homology of this protein family, overlapping and unique functions of the individual FXPs have been reported. In general, the FXPs are known to be associated with polyribosomes acting as regulators of protein synthesis involved in transport and translational suppression of target mRNAs, thereby regulating local translation, e.g., at synapses in neurons important for synaptic plasticity and excitability of neurons. However, additional functions of the FXPs have emerged. In RNA metabolism, FMR1 and FXR1 are involved in ADAR-mediated RNA editing, and this modification may play a role in recognition of FMR1/FXR1 target mRNAs. Additionally, the FXPs may be involved in transcription, splicing, and nuclear export of mRNAs, as well as in mRNA stability. Therefore, spatiotemporal regulation and tuning of gene expression is likely one of the main functions of the FXPs [3, 4]. However, functions of the FXPs in RNA metabolism are not limited to mRNAs. Association of the FXPs with the miRNA machinery is well established and thought to support translational silencing of targeted mRNAs via the RNA-induced silencing complex (RISC) [12]. The finding that the FXPs directly interact with a subset of miRNAs, without the involvement of Ago proteins, may be indicative for additional/distinct mechanisms [6]. Much less is known about the relation of the FXPs to long noncoding (lnc) RNAs that are particularly abundant in the brain. However, here too, increasing evidence points towards an involvement of the FXPs in lncRNA biology (e.g., [13]).

**Fig. 2:**
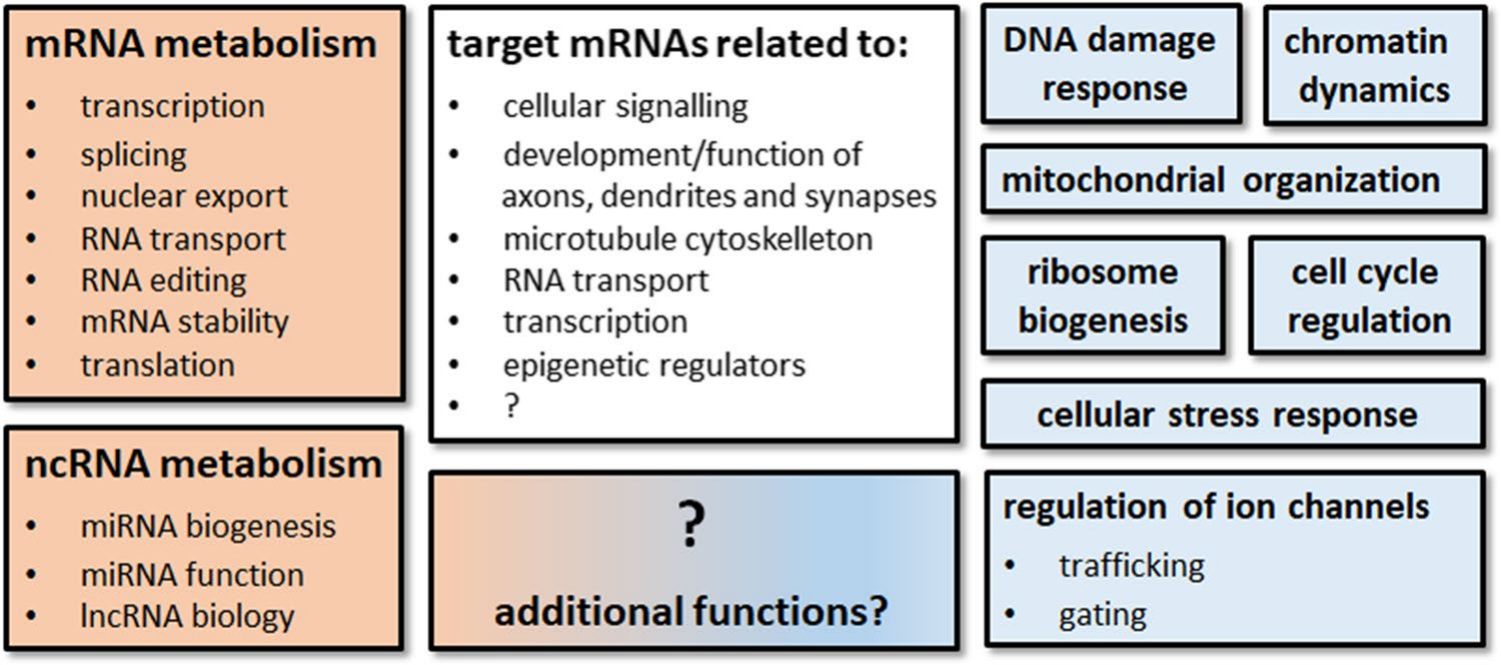
Overview of FXP functions. Summary of cellular processes that have been linked to the FXPs. Functions mainly dependent on the RNA-binding properties of these proteins are shown in orange. Blue boxes indicate involvement of the FXPs in cellular pathways putatively by protein-protein interactions. The white box shows functions of FXP target mRNAs. Please note that most FXP functions shown here have been identified by studying FMR1, and it is not yet known if FXR1 and FXR2 are involved in all processes shown here

Other functions of the FXPs beyond RNA metabolism are their involvement in chromatin dynamics and the DNA damage response, cell cycle regulation, ribosome biogenesis, and mitochondrial organization [3, 4, 14]. In neurons, FMR1 is an important regulator of ion channels at multiple levels. Besides regulating local translation of mRNAs coding for diverse ion channels, FMR1 is additionally involved in channel’s trafficking and gating via protein-protein interactions [15]. Furthermore, the FXPs play an important role in cellular stress responses and stress granule formation. Stress granules are membrane-less accumulations of RNAs and proteins forming in the cytoplasm by phase separation of proteins/RNAs, and promoting survival of most cell types under conditions of stress [16]. While it is known that all three FXPs, similar to many other RBPs, are components of stress granules [17, 18], FMR1 is essential for stress granule assembly [19]. Interestingly, different stressors in neuronal and non-neuronal cell types induce expression of FMR1, further substantiating its role in cellular stress responses. Here, it is worth mentioning that lower and higher expression levels of FMR1 are associated with decreased and increased cell viabilities, respectively [14, 20, 21]. The role of FXR1 and FXR2 in stress granule dynamics has not been studied in detail yet. Figure 2 summarizes the various functions of the FXPs.

### Post-translational Modifications

Regarding the multiple functions and cellular locations, it is not surprising that the FXPs are regulated by various post-translational modifications (PTMs). These include phosphorylation, methylation, acetylation, sumoylation, and ubiquitylation [3, 22, 23]. However, the enzymes and signaling pathways involved, as well as functional consequences of these PTMs, are largely unknown. The best-studied PTM of the FXPs is the phosphorylation of serine 500 (S500 in humans, S499 in mice) of FMR1 that is likely mediated by the constitutive active casein kinase II (CK2), and required for its role in regulating translation. Only phosphorylated FMR1 associates with polyribosomes and RISC resulting in translational repression of target mRNAs. Dephosphorylation of FMR1 by protein phosphatase 2A (PP2A) leads to its release from ribosomes and Ago2 facilitating translation. Phosphorylation of S500 has also been shown to promote phase separation of FMR1 required for the formation of different RNA granules involved in RNA transport and stress responses. Release of FMR1 from RNA granules is likely induced by sumoylation. Dephosphorylation of S500 is additionally a prerequisite for the ubiquitylation of FMR1 and its degradation via the ubiquitin-proteasome-system (UPS). The UPS has also been shown to be involved in the degradation of FXR1, but by a different mechanism and in another context [3, 23]. Another PTM of FXR1 and likely of FMR1, but not of FXR2, is phosphorylation of S420 of FXR1 and the conserved residue (S511) of FMR1 by the multifunctional kinase PAK1. At least in zebrafish muscle development, this PTM is essential for FXR1 function. Furthermore, this PTM increases when FXR1/FMR1 are recruited to arsenite-induced stress granules, indicating a role of this PTM in cellular stress responses [24]. The RGG domains of FMR1 and FXR1 may be methylated affecting their association with polyribosomes and their capability to form homo- and heterodimers with other FXPs. While methylation of FXR1 may decrease its RNA-binding activity in general, differential mRNA-binding is observed for FMR1 depending on the methylation state of specific arginines [3, 23]. Causes and consequences of multiple additional PTMs of the FXPs detected by proteome-wide approaches [22] have not been studied yet. Unfortunately, there is almost no functional data available about the PTMs of FXR2.

### FXP-Associated Hereditary Diseases

The majority of genetic diseases linked to the FXPs are associated with severe developmental defects. Deficiency or absence of FMR1 protein causes FXS, which is the most common cause of inherited intellectual disability and ASDs. The primary cause of FXS is a trinucleotide (CGG) repeat expansion in the promoter region of *FMR1* leading to hypermethylation and silencing of expression. Many of the various symptoms of FXS are attributed to the multiple functions of FMR1 and/or its mRNA targets in development and maintenance of synapses. While healthy individuals have ≤ 44 CGG repeats, > 200 repeats are pathogenic and cause silencing of *FMR1*, and consequently FXS. Intermediate repeat lengths (i.e., 55–200) are linked to two other diseases, namely, fragile X–associated premature ovarian insufficiency (FXPOI) in women and, predominantly in men, fragile X–associated tremor/ataxia syndrome (FXTAS) [3, 25]. FXPOI is the most frequent cause of monogenic premature ovarian insufficiency, while FXTAS is a late-onset neurodegenerative disease. Interestingly, in contrast to FXS, in both FXPOI and FXTAS, expression of FMR1 protein is not or only mildly decreased, and increased transcription of repeat-containing *FMR1* mRNA sequestering RBPs and/or being translated into aberrant FMR1 protein may represent the dominating pathogenic mechanism [26, 27]. Recently, diseases associated with intermediate *FMR1* CGG repeat lengths have been extended by the fragile X–associated neurodevelopmental disorders (FXANDs) that usually include anxiety and depression [28].

Besides FMR1, also FXR1 is linked to developmental diseases, namely, congenital myopathies that differ in severity depending on the specific mutation. These diseases underly recessive mutations in muscle-specific exon 15 of *FXR1*. The milder form (proximal, with minicore lesions [MYOPMIL]) is a result of strongly reduced expression of FXR1, while the very severe form (with respiratory insufficiency and bone fractures [MYORIBF]) is due to expression of a mutant protein prone to aggregation [29]. Additionally, genome-wide association studies repeatedly linked *FXR1* to schizophrenia and bipolar disorders [3]. So far, no hereditary diseases associated with *FXR2* have been reported.

## The FXPs in ALS Pathogenesis

When considering the multiple functions and mRNA targets of the FXPs, it is not too surprising that these proteins are involved in cellular processes and pathways impaired in ALS. However, compared to other RBPs that do not cause ALS but may contribute to the pathogenesis, e.g., some members of the heterogenous nuclear ribonucleoprotein (hnRNP)-family [30], available evidence suggests a prominent role of the FXPs.

### Regulation of Transport and Translation of Target mRNAs

Cytoplasmic mis-localization and aggregation of RBPs with important functions in RNA metabolism, such as TDP-43 and FUS, are a neuropathological hallmark of most ALS cases. Neuronal cytoplasmic inclusions (NCIs) containing abnormally phosphorylated and fragmented TDP-43 are found in affected neurons and some other cell types of ≈97% of all ALS patients, largely independent of the underlying cause of the disease [1, 2]. Moreover, rare variants in *TARDBP* coding for TDP-43 are causative for ALS [31] indicating a central role of this RBP in ALS pathogenesis, beyond the possibility of being a simple marker for dying neurons. FUS is another RBP that, similar to TDP-43, shows cytoplasmic mis-localization and aggregation in affected tissues of ALS patients. However, at least in ALS, FUS pathology is most prominent in patients carrying a pathogenic *FUS* variant [1, 32], but has also been detected in sALS patients [33, 34]. It is worth to mention that most pathogenic mutations in *FUS* are very aggressive and associated with an early-onset and a fast disease progression [35]. FALS patients with pathogenic mutations in *SOD1* are exceptions to the almost universal TDP-43 and/or FUS pathology found in ALS. They also show abnormal protein aggregation in affected tissues, but these aggregates contain predominantly SOD1 protein [32].

#### Functional Relationship of the FXPs and TDP-43 in Regulating Local Translation

The finding that FMR1 and TDP-43 co-localize in RNA granules [36, 37] and cooperatively regulate transport and local translation of some shared target mRNAs [37–39] led to the idea of a partial, age-related redundancy of these proteins. In this model, FMR1 is important in development, and loss of/reduced expression or malfunction of this protein is rather associated with neurodevelopmental disorders. TDP-43, on the other hand, is more important in adulthood and/or ageing. Here, pathogenic mutations are linked to neurodegenerative diseases [40]. Indeed, by combining several studies aiming at identifying target mRNAs of FMR1 or TDP-43, 1140 mRNAs were found binding to both proteins. However, cooperative regulation of transport and translation of these mRNAs that include ≈160 mRNAs important for neuronal development, structure, and function has not been demonstrated yet [39]. Furthermore, when considering the problems associated with FMR1 target mRNA identification (see above), reliability of the 1140 mRNAs bound by both proteins is questionable, and further validation is required. Therefore, the extent of redundancy of FMR1 and TDP-43 remains to be determined. Nevertheless, cooperative regulation of selected mRNAs, including some that are highly important in neurons, has been shown. These include the mRNAs of *SIRT1* [37], *Rac1*, *GluR1*, and *Map1b* [38, 39]*.*

Highly interesting results regarding a link of TDP-43 and FMR1 in the context of ALS were achieved in an in vivo Drosophila model. Here, overexpression of both TDP-43 wild-type or an ALS-associated variant (G298S) induced a depigmentation phenotype of the eye that indicates neurodegeneration in the neuroepithelium, increased larval turning times and severely reduced life span. In both cases, overexpression of the single *Drosophila* homolog of the FXP family, dFMR1, fully or partly rescued these phenotypes. In contrast, RNAi-mediated knockdown or genetic depletion of dFMR1 worsened the TDP-43-induced phenotypes. Importantly, overexpression of dFMR1 did not rescue increased larval turning times induced by knockdown of TBPH (the *Drosophila* homolog of TDP-43). Hence, this model rather argues against profound redundancy of TDP-43 and FMR1, but undoubtedly identifies the FXPs as important modifiers of TDP-43-induced neurotoxicity. It is noteworthy that dFMR1 rescued the TDP-43 associated phenotypes at multiple levels including restoration of *futsch* (the *Drosophila* homolog of *MAP1B*) mRNA translation and of neuromuscular junction pathology, as well as by decreasing TDP-43 aggregation [41].

Very little is known about the relation of FXR1 and FXR2 to TDP-43. However, considering that both proteins interact with TDP-43 [7] and are present in a fraction of FMR1-containing RNA granules [42], a functional relationship similar to FMR1 is likely but has not been demonstrated yet.

#### Mutant FUS Impairs FXP Function

First evidence for a possible involvement of the FXPs in FUS-ALS was provided by the finding that FUS physically interacts with all three FXPs [7]. Here, the interaction of FUS and FMR1 (see above) is direct [7, 8], while the interaction with FXR1 and FXR2 is much less pronounced, at least when focusing on the TADs of the FXPs [6]. Furthermore, the FXPs have repeatedly been shown to localize to cytoplasmic FUS granules formed by ALS-associated FUS variants in both non-neuronal cells and iPSC-derived motoneurons [6, 8, 43]. Importantly, when considering that both FUS and the FXPs are components of stress granules [17, 18, 44], this co-localization was evident without induction of stress. Interestingly, expression level of at least FMR1 may be linked to the nucleocytoplasmic distribution of FUS, because in HEK293T cells knockdown of FMR1 led to nuclear retention of both wild-type and mutant FUS [8].

**Fig. 3:**
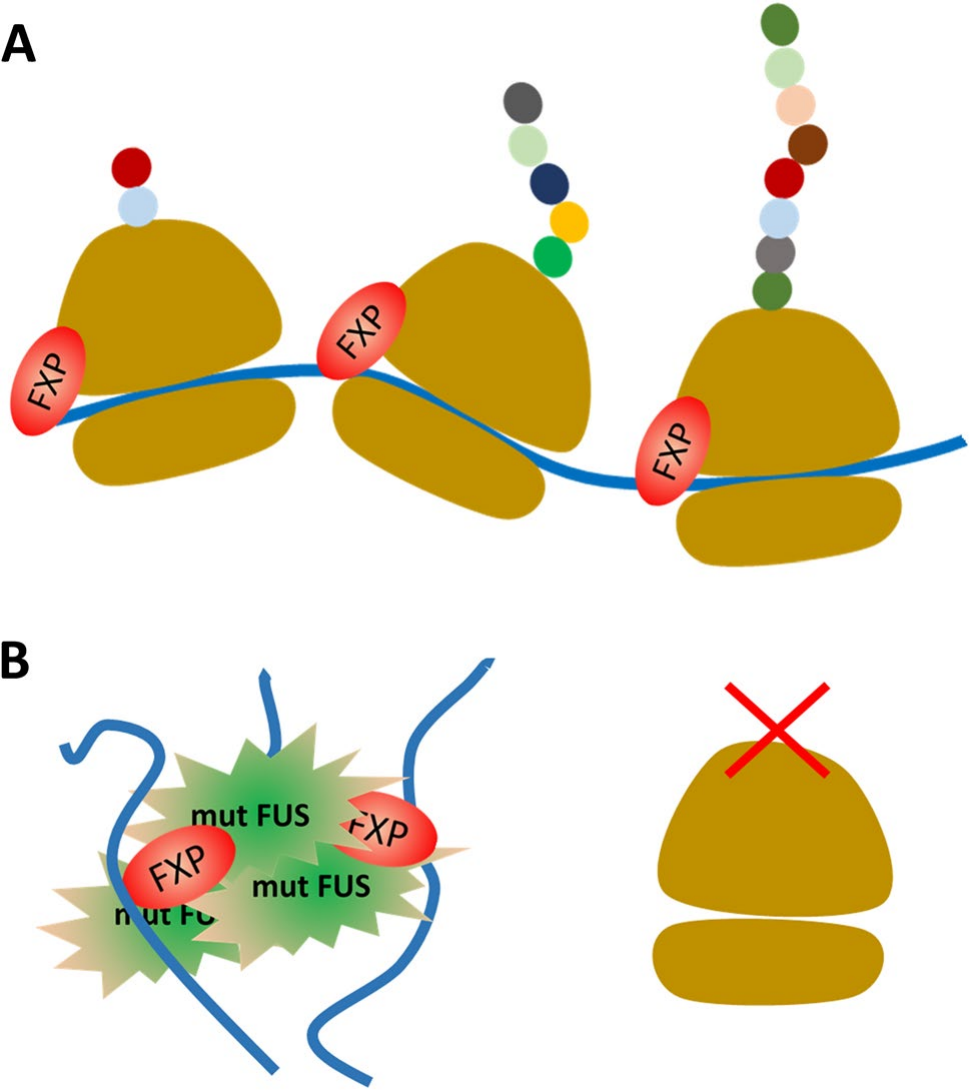
Contribution of the FXPs to FUS-linked ALS. In healthy individuals (**A**), the FXPs are associated with polyribosomes and regulate translation of various target mRNAs. In motoneurons of individuals suffering from FUS-linked ALS (**B**), aggregates of mutant FUS sequester the FXPs including bound mRNAs resulting in decreased translation of FXP target mRNAs

Recently, elegant studies revealed a role of FUS in translation. Besides multiple additional functions, FUS can, similar to the FXPs, associate with polyribosomes and is involved in translational regulation. ALS-associated variants of FUS have been shown to repress protein synthesis globally [45–48]. Mechanistically, it has been proposed by polysome profiling that ALS-related mutations lead to an increased association of FUS with ribosomes (ribosomal subunits, monosomes, and polysomes) resulting in increased repression of translation [47]. However, binding of FUS to ribosomes was not measured directly, and increased presence of mutated FUS in ribosomal fractions may reflect increased aggregation propensity. Indeed, another study showed decreased association of mutant FUS with polysomes [43]. Here, the authors suggest a mechanism of increased translational repression involving FMR1. They convincingly show that wild-type FUS promotes the association of FMR1 with the translational machinery, and that mutant FUS sequesters FMR1 in FUS condensates. Importantly, in this model, FMR1 is removed from polysomes by mutant FUS including bound mRNAs resulting in decreased translation of FMR1 target mRNAs (Fig. 3). Comparing the ribosome-bound mRNAs (translatome) from control and mutant FUS mice indeed confirmed depletion of FMR1 target mRNAs [43]. However, here too, due to the problems associated with FMR1 target mRNA identification (see above), these results have to be interpreted with care.

Another interesting mechanism involving FMR1 in FUS-linked ALS has recently been explored in iPSC-derived motoneurons. In this scenario, FMR1 represses the translation of the RBP HuD (encoded by *ELAVL4*). Mutant FUS mis-localizing to the cytoplasm competes with FMR1 for binding to the HuD mRNA resulting in increased expression of HuD protein. Increased HuD protein, in turn, leads to abnormal stabilization of target mRNAs coding for GAP43 and NRN1, and consequently higher protein levels. Here, knockdown of NRN1 was sufficient to partially but substantially rescue an axonal branching phenotype induced by mutant FUS [49].

Most likely the best evidence indicating an involvement of the FXPs in ALS caused by mutant FUS is, similar to TDP-43, provided by an in vivo model. Here, in zebrafish, it has been shown that phenotypes induced by overexpression of mutant FUS (R521C), such as loss of neuromuscular junction integrity and aberrant touch-evoked escape responses (TEERs), are fully rescued when FMR1 is overexpressed along with mutant FUS. Mechanistically, the authors have shown that mutant FUS leads to an increase of Map1b protein in synaptosomes that is normalized by FMR1 at the level of translation [7]. Unfortunately, additional targets and mechanisms were not addressed in this study.

As for TDP-43, there is almost no data available about a possible relation of FXR1 and FXR2 to FUS. However, similar to FMR1, also FXR1 and FXR2 are sequestered in cytoplasmic granules containing mutant FUS [6] suggesting a contribution of these proteins to ALS pathogenesis that awaits experimental testing.

### FXPs and Pathogenic Protein Aggregation

Besides mounting evidence that ALS-related proteins, such as TDP-43 and FUS, impact FXP functions, there are hints in the literature that the FXPs may be implicated in a hallmark of ALS, namely, the pathogenic aggregation of specific proteins in affected tissues [1, 2].

In ALS post-mortem tissue, available data are limited, but it has been shown that FMR1 localizes to NCIs of wild-type TDP-43 in sporadic ALS, as well as of mutant FUS in FUS-linked ALS [7]. Additionally, FXR1, but not FXR2, is a component of some (≈20%) FUS inclusions. Interestingly, aberrant expression of FXR1 and FXR2 in ALS spinal cord motoneurons was evident independent of the underlying cause of the disease, at least in *FUS*- and *C9orf72*-linked fALS, as well as in sALS. Here, compared to the homogenous expression in controls, ≈15–40% of motoneurons showed higher level of FXR1 and FXR2, while substantially lower levels of these proteins were evident in ≈5–20% of motoneurons. Roughly 25–50% of motoneurons with high FXR1 or FXR2 expression additionally showed accumulations or aggregates of these proteins. Strikingly, at least in *FUS*-linked ALS, low expression of FXR1 and especially FXR2 correlated with the presence of FUS NCIs [6].

Mechanistically, it has been repeatedly shown that the FXPs are sequestered by cytoplasmic condensates spontaneously formed by mutant FUS impacting FXP functions (see above). However, an inverse scenario, i.e., that FXP containing granules may sequester cytoplasmic FUS, and serve as hubs for FUS aggregation, has never been addressed. For TDP-43, there are some hints that this may indeed be the case. It has been shown that cytoplasmic TDP-43, induced either by overexpression of specific variants in cell lines [50] or by traumatic brain injury in mice [51], partly localizes to FMR1 containing granules that are not necessarily stress granules. In cell lines, additional application of stress (sodium arsenite) was required for maturation of permanent TDP-43 aggregates separating from FMR1 containing stress granules [50]. In mice, these cytoplasmic TDP-43/FMR1 granules were reversible and did not mature to permanent aggregates, even when mice expressing an ALS-associated variant of TDP-43 (G298S) were used for the experiments [51]. Therefore, it remains to be determined if FXP containing granules are implicated in ALS-related protein aggregation. Nevertheless, the finding that overexpression of dFMR1 mitigated the phenotype of a TDP-43-linked *Drosophila* ALS model possibly by reducing TDP-43 aggregation argues for a contribution of the FXPs [41], either directly or indirectly via regulation of other factors.

Recently, a study not related to ALS provided the basis for another hypothesis, how the FXPs may contribute to the aggregation of TDP-43. Here, knockdown of the individual FXPs in non-neuronal cell lines induced the formation of cytoplasmic condensates of nuclear pore components [52]. Considering that TDP-43 co-aggregates with components of the nuclear pore [53, 54], and nuclear pore pathology may even precede mis-localization and aggregation of TDP-43 [55], it is tempting to hypothesize that the FXPs are involved in this process. Findings in ALS post-mortem tissue indicating restriction of NCIs to motoneurons with low expression level of the FXPs [6] would be in line with this hypothesis, but there is no experimental evidence yet.

### FXPs and ALS-Related miRNAs: an Indicator for Presymptomatic FXP Dysfunction?

MiRNAs are generally known to fine-tune gene expression post-transcriptionally by recruiting RISC to more or less complimentary sequences in the 3′ untranslated region of target mRNAs, resulting in degradation and/or translational repression [56]. In ALS, miRNAs are of special interest, because several causative genes, such as *TARDBP* [57, 58]*, FUS* [59]*, HNRNPA1* [60]*, HNRNPA2B1,* or *MATR3* [61], have been linked to miRNA biogenesis and/or function, and even a general defect of miRNA biogenesis is discussed [62]. Additionally, genetic ablation of the endoribonuclease catalyzing the final cleavage of miRNA-precursors to mature miRNAs, *Dicer1*, from spinal motoneurons of mice leads to an ALS-like phenotype [63], further pronouncing importance of miRNA-mediated regulation of gene expression for motoneuron survival.

With regard to the FXPs, we could show that all three directly interact with a specific subset of mature miRNAs [6]. These ALS-related miRNAs are highly enriched for a sequence motif (GDCGG; D = G, A, or U), and were previously found downregulated in serum samples of fALS patients with different genetic causes (*FUS*, *SOD1,* and *C9orf72*) of the disease [64], as well as in the majority (>60%) of sALS patients [65]. Additionally, the FXPs are involved in the biogenesis and/or degradation of these ALS-related miRNAs [6], whereby specific mechanisms, functions, and associated consequences have not been explored yet. Our finding that this subset of miRNAs was already downregulated in serum of presymptomatic carriers of causative ALS mutations (*SOD1*, *C9orf72,* and *PFN1*; [64]), as well as in FUS mutant iPSC-derived motoneurons that most likely represent a presymptomatic or early stage of the disease, may indicate an involvement of the FXPs in very early pathogenic mechanisms. Unfortunately, despite multiple studies addressing miRNAs in ALS, downregulation of the FXP-related GDCGG-miRNAs was not confirmed by other groups yet. This is, most likely, due to the fact that we used microarrays for detection, and these miRNAs are poorly covered by small RNA sequencing applied in most studies [6]. Nevertheless, our results, at least at the level of specific miRNAs, point towards converging disease mechanisms in ALS with different underlying causes that may involve the FXPs.

### FXPs and Defects in RNA Editing

It is known for a long time, that, predominantly in the CNS, mRNAs coding for subunits of α-amino-3-hydroxy-5-methyl-4-isoxazole propionic acid (AMPA) receptors may be post-transcriptionally modified leading to amino acid substitutions at critical sites associated with channel permeability [66]. The finding that editing efficiency of such a critical site (commonly referred to as Q/R site) of the GluA2 subunit (encoded by *GRIA2*) of AMPA receptors in laser-dissected motoneurons from ALS patients is markedly reduced, implicated defective RNA editing in ALS pathogenesis [67]. Unedited Q/R sites of GluA2 are associated with increased Ca^2+^ influx that has been linked to multiple features of ALS including hyperexcitability, excitotoxicity, mis-localization of TDP-43, and death of motoneurons [68]. Besides this specific editing site, a recent study focusing on *C9orf72*-related ALS/FTD revealed widespread disease-associated RNA editing aberrations in different post-mortem brain regions as well as in iPSC-derived motoneurons. Most likely, these changes were induced by the cytoplasmic mis-localization of the RNA editing enzyme ADAR2 [69].

Interestingly, the FXPs, and especially FMR1 and FXR1, are closely linked to RNA-editing pathways. Elegant experiments in *Drosophila* showed that the homolog of the FXP family, dFMR1, physically interacts with and modulates the activity of dADAR, an A-to-I RNA-editing enzyme. Here, loss or overexpression of dFMR1 led to deregulated RNA editing especially of mRNAs involved in synaptic transmission and neuromuscular junction architecture [70]. Similar results were reported in zebrafish [71], mice [72], and post-mortem brains of humans suffering from ASDs [73]. In humans, FMR1 interacts with the catalytically active A-to-I RNA editing enzymes ADAR1 and ADAR2 (encoded by *ADAR* and *ADARB1*, respectively), while FXR1 interacts with ADAR1 only. The finding that, in ASDs, sites of differential RNA editing are in close proximity to FMR1- and FXR1-binding sites suggest that the FXPs are directly involved in recruiting ADAR enzymes to mRNA-editing sites. Interestingly, while FMR1 promotes editing of specific mRNA sites, FXR1 was found to repress editing [73]. So far, it has not been explored if the differential RNA editing and/or the cytoplasmic mis-localization of ADAR2 reported in *C9orf72*-linked ALS [69] may be related to deregulated FXPs. In HeLa cells, knockdown of FMR1 or FXR1 did not induce cytoplasmic mis-localization of ADAR1 and/or ADAR2 [73]. Regarding the ALS-related Q/R site of GluA2 (see above), it is not known if the FXPs are involved in insufficient editing. However, presence of GluA2 mRNA in FMR1-containing granules [74], as well as ASD-like phenotypes of carriers of GluA2 Q/R site mutations [75], indicate involvement of FMR1. Nevertheless, aberrant expression of FXR1 in spinal cord motoneurons of ALS patients [6], or sequestration of FMR1 in NCIs of TDP-43 or FUS [7], will most likely affect RNA editing, including possible consequences on synaptic transmission, neuromuscular junction integrity, and motoneuron survival.

### Additional Hints Indicating a Contribution of the FXPs to ALS Pathogenesis

In addition to the studies discussed above, there are several findings published that may relate the FXPs to ALS pathogenesis, but have not been explored in detail yet. For example, besides binding to TDP-43 and FUS, all three FXPs interact with Ataxin-2 (encoded by *ATXN2*) [7], an important risk gene [76] and modifier of ALS [77]. FMR1 and FXR2 interact with the protein product of another established ALS gene, *TBK1* [78, 79]. If this interaction is associated with the phosphorylation of critical sites of FMR1 and/or FXR2 is not known. Furthermore, FMR1 positively regulates the translation of *SOD1* mRNA [80], and proteomics identified deregulation of some FMR1 targets in spinal cord synaptoneurosomes in SOD1 G93A mice [81].

In *C9orf72*-linked ALS, all three FXPs have repeatedly been shown to interact with repeat expansion-containing RNA [82–85], and at least FMR1 localizes to repeat expansion induced RNA foci [85]. Additionally, in a *Drosophila* model of *C9orf72* repeat expansions, FMR1 modulated dendritic branching defects, and increased level of FMR1, as well as of an established FMR1 target (PSD-95), were detected in human *C9orf72* mutant iPSC-derived motoneurons. Furthermore, transcriptomics of *C9orf72* mutant human post-mortem cortex revealed an enrichment of FMR1 target mRNAs among downregulated mRNAs [86].

**Fig. 4:**
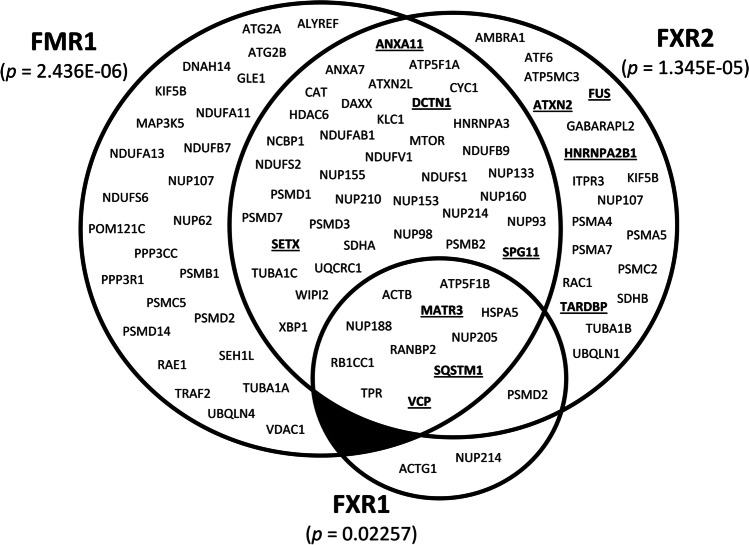
Target mRNAs of the FXPs are related to ALS. Venn diagram showing target mRNAs of the individual FXPs in non-neuronal cells (see text for details) related to the KEGG pathway ‘Amyotrophic Lateral Sclerosis’. *P*-values (adjusted) correspond to enrichment of ALS-related mRNAs among all target mRNAs and were calculated using the Enrichr database [92]. The mRNAs of causative ALS genes are highlighted

Last but not least, also target mRNAs of the FXPs indicate implication of these proteins in ALS pathogenesis. The Encyclopedia of DNA Elements (ENCODE; [87]) dataset of RNA-protein interactions generated by enhanced UV cross-linking immunoprecipitation (eCLIP; [88]) allows comparison of target mRNAs of the individual FXPs. Strikingly, according to the Kyoto Encyclopedia of Genes and Genomes (KEGG; [89]) target mRNAs of FMR1 and FXR2 are highly significantly enriched in genes related to ALS, including some causative ALS genes. Enrichment of FXR1 targets is much less pronounced, but still significant (Fig. 4). These interactions include, but are not limited to, mRNAs of definite ALS genes *TARDBP*, *FUS* (FXR2), and *VCP* (all three FXPs), as well as the clinical modifier *ATXN2* (FXR2; classification of ALS genes according to [90, 91]). However, this relation has to be interpreted with care, because the ENCODE dataset is derived from non-neuronal cells (HepG2 and K562), and because of the problems associated with FXP target mRNA identification mentioned above. Nevertheless, at least some of the interactions are likely of relevance in neurons, and in ALS pathogenesis.

## The FXPs in Other Age-Related Neurodegenerative Diseases

Although this review focuses on available evidence for a contribution of the FXPs to ALS pathogenesis, it is worth mentioning that these proteins have been implicated in two other age-related neurodegenerative diseases, namely, Alzheimer’s disease (AD) and Parkinson’s disease (PD). Strikingly, similar to ALS, downregulation of FXP family members has been linked to early pathogenic events, which precede or perhaps even cause the deposition of aggregates.

In AD, FMR1 suppresses the translation of *APP* mRNA and loss of/reduced expression of FMR1 may contribute to *APP* overexpression and deposition of amyloid plaques [93]. Indeed, decreased FMR1 expression was evident in pre-symptomatic and early symptomatic, but not in fully symptomatic, AD model mice [94]. In PD, a recent study employing cell culture experiments, mouse models, and human *post-mortem* brain tissue convincingly showed that FMR1 is downregulated in vulnerable neurons, and that reduced FMR1 protein most likely precedes Lewy body pathology. Moreover, the authors found that overexpression of α-synuclein induces downregulation of FMR1 [95]. Notably, in serum of PD patients, we found downregulation of GDCGG-miRNAs [96] reminiscent of our findings in ALS (see above). These data expand the relevance of the FXP family from ALS with different underlying causes also to AD and PD and emphasize overlap of at least some pathogenic processes in different neurodegenerative diseases.

## Conclusion and Future Perspectives

In summary, a number of excellent studies leave little doubt about an involvement of the FXP family in ALS pathogenesis. Here, the most convincing data, including analyses of human materials, animal models, and mechanistic studies, are available for the relation of the FXPs to FUS- and TDP-43-linked ALS. Additionally, there are many hints in the literature that the FXPs may play a role in other genetic forms of ALS linked to *SOD1*, *PFN1, C9orf72, ATXN2,* and *TBK1,* as well as in sALS. This list could even be expanded when including mRNAs of ALS genes putatively binding to one of the FXPs (see Fig. 4). Therefore, reduced expression and/or loss-of-function of the FXPs may represent a pathogenic event in ALS and argues for the existence of converging disease mechanisms evident in most, if not all, patients. However, further studies are required to elucidate importance of these findings for human ALS. Mutations in different genes likely have different effects on the FXPs, but may converge on a single outcome, namely, reduced FXP expression and/or function. Despite large-scale studies (e.g., [97]), not any of the FXPs has been genetically linked to ALS so far. This may be explained by the fact that these proteins have important functions in development and are rather associated with developmental than age-related diseases (see above). Therefore, it is more likely that loss of function and/or expression of the FXPs in ALS represents one of several “hits” necessary for developing ALS, as suggested in multistep models of ALS [98, 99].

Considering that deregulation of the FXPs is most likely an early or even presymptomatic event in ALS pathogenesis, these proteins, or upstream events affecting these proteins, may represent promising novel therapeutic targets. However, there are still many open questions requiring clarification. Most importantly, (i) there is a strong bias in studying FMR1 in ALS while FXR1 and FXR2 are largely neglected. Is one of the FXPs of special importance, or do all three FXPs equally contribute to the disease? Here, comparative studies are required, especially because of the possibility that different FXPs may be of relevance in ALS patients with distinct underlying causes. (ii) Overexpression of FXPs substantially or even fully rescued phenotypes of *Drosophila* and zebrafish models of ALS. Is this finding also true in mammals? Clarifying this question in well-defined ALS mouse models would provide better estimates of potential benefits for human patients. (iii) How do the FXPs behave during the course of the disease? At what timepoint occurs deregulation of each individual FXP? These questions should be addressed in different genetic mouse models to determine optimal timepoints of intervention, and to better understand the sequence of pathogenic events. (iv) Many of the studies described above linked the FXPs to protein aggregation. Are the FXPs implicated in additional pathogenic mechanisms? When considering the multiple functions of these proteins, they may additionally be involved in other ALS-related processes, e.g., mitochondrial dysfunction.

There is still a long way to go regarding the FXPs in ALS and other neurodegenerative diseases, but results available so far are promising. In contrast to targeting expression of mutated genes using antisense oligonucleotides or alternative approaches, targeting the FXPs may result in treatment options for most patients, including those without a genetic mutation in a common causative gene. While it is rather unlikely that restoring FXP function and/or expression will cure the respective disease, every improvement compared to therapies available now is a success.

## Data Availability

Not applicable.
